# Levosimendan Plus Dobutamine in Acute Decompensated Heart Failure Refractory to Dobutamine

**DOI:** 10.3390/jcm9113605

**Published:** 2020-11-09

**Authors:** William Juguet, Damien Fard, Laureline Faivre, Athanasios Koutsoukis, Camille Deguillard, Nicolas Mongardon, Armand Mekontso-Dessap, Raphaelle Huguet, Pascal Lim

**Affiliations:** 1 Department of Cardiovascular Medicine, AP-HP, Henri Mondor University Hospital, 94000 Créteil, France; damien.fard@gmail.com (D.F.); laureline.faivre@aphp.fr (L.F.); ath.koutsoukis@gmail.com (A.K.); Camille.deguillard@aphp.fr (C.D.); raphaelle.huguet@aphp.fr (R.H.); lim.pascal.hmn@gmail.com (P.L.); 2 Institut Mondor de Recherche Biomédicale, INSERM U955, Université Paris-Est Créteil, 94000 Créteil, France; armand.dessap@aphp.fr; 3 Department of Surgical Intensive Care, AP-HP, Henri Mondor University Hospital, 94000 Créteil, France; nicolas.mongardon@aphp.fr; 4 Department of Medical Intensive Care, AP-HP, Henri Mondor University Hospital, 94000 Créteil, France

**Keywords:** acute decompensated heart failure, levosimendan, dobutamine, vena cava oxygen saturation

## Abstract

Randomized studies showed that Dobutamine and Levosimendan have similar impact on outcome but their combination has never been assessed in acute decompensated heart failure (ADHF) with low cardiac output. This is a retrospective, single-center study that included 89 patients (61 ± 15 years) admitted for ADHF requiring inotropic support. The first group consisted of patients treated with dobutamine alone (*n* = 42). In the second group, levosimendan was administered on top of dobutamine, when the superior vena cava oxygen saturation (ScVO2) remained <60% after 3 days of dobutamine treatment (*n* = 47). The primary outcome was the occurrence of major cardiovascular events (MACE) at 6 months, defined as all cause death, heart transplantation or need for mechanical circulatory support. Baseline clinical characteristics were similar in both groups. At day-3, the ScVO2 target (>60%) was reached in 36% and 32% of patients in the dobutamine and dobutamine-levosimendan group, respectively. After adding levosimendan, 72% of the dobutamine-levosimendan-group reached the ScVO2 target value at dobutamine weaning. At six months, 42 (47%) patients experienced MACE (*n* = 29 for death). MACE was less frequent in the dobutamine-levosimendan (32%) than in the dobutamine-group (64%, *p* = 0.003). Independent variables associated with outcome were admission systolic blood pressure and dobutamine-levosimendan strategy (OR = 0.44 (0.23–0.84), *p* = 0.01). In conclusion, levosimendan added to dobutamine may improve the outcome of ADHF refractory to dobutamine alone.

## 1. Introduction

Acute decompensated heart failure (ADHF) with low cardiac output and cardiogenic shock (CS) results from a severe imbalance between oxygen supply and consumption. The mortality remains high, especially when the imbalance is not corrected. In patients with CS, the balance between oxygen supply and consumption can be assessed using the superior vena cava oxygen saturation (ScVO2), aiming at a target value of >60%. Inotropic agents are the first-line treatment, in the effort to increase cardiac output and restore oxygen supply. Dobutamine remains the first choice because of its availability and low cost. However, dobutamine’s hemodynamic efficacy is often inadequate or non-sustained [1,2,3]. Levosimendan is a calcium sensitizer agent which enhances cardiac contractility. Several studies have reported that the use of levosimendan in acute decompensated heart failure is associated with an improvement of hemodynamic parameters and cardiac function but its beneficial effect on survival and mortality remains debated [4,5,6]. To overcome these limitations, Nanas and al. [7] suggested adding levosimendan to dobutamine in order to obtain a synergic inotropic effect. In patients with decompensated heart failure (*n* = 18), the authors reported a greater improvement in cardiac index and pulmonary capillary wedge pressure when levosimendan was added to dobutamine. We hypothesized that this strategy may be beneficial in patients with acute decompensated heart failure with low cardiac output refractory to dobutamine.

## 2. Methods

### 2.1. Population

The study retrospectively included patients admitted for ADHF at Henri Mondor Hospital (from September 2013 to December 2017). ADHF with low cardiac output was defined by the following criteria: (1) cardiac index < 2.2 L/min/m^2^, (2) elevated right or left ventricular (LV) filling pressures, (3) low ScVO2 (< 60%), AND (4) signs of impaired organ perfusion (renal failure, hepatic failure or arterial blood lactate elevation) OR systolic blood pressure < 90 mmHg or mean arterial blood pressure < 65 mmHg. ADHF related to cardiac surgery, sepsis, cardiac amyloidosis, acute myocarditis, valvular heart disease or acute coronary syndromes were excluded to avoid bias related to the outcome. The population was divided in two groups. The historical group (dobutamine group, from 2013 to 2015) included consecutive patients with ADHF managed with dobutamine alone. In the second group (dobutamine-levosimendan, from 2015 to 2017) levosimedan infusion was administered as an adjunctive treatment in patients refractory to dobutamine, defined as superior vena cava oxygen saturation (ScVO2) < 60% after 3 days of dobutamine treatment. Study protocol was approved by Henri Mondor University Hospital’s ethics committee (registration code n°1778041) and informed consent was obtained from all included patients.

### 2.2. Cardiac Assessment and Hemodynamic Monitoring

All patients admitted for ADHF underwent transthoracic echocardiography (TTE) (Vivid E95^®^ or Vivid S70^®^, GE Vingmed Ultrasound, Horten, Norway) to assess left ventricular ejection fraction (LVEF), right ventricular systolic function and valvular disease. Cardiac output was assessed by TTE using the left ventricular outflow tract diameter in parasternal long axis view and left ventricular outflow tract velocity time integral (LVOT VTI) by pulsed Doppler (Vivid E95^®^ or Vivid S70^®^, GE Vingmed Ultrasound, Horten, Norway) in five chamber view. American Society of Echocardiography (ASE) and European Association of Cardio-Vascular Imaging (EACVI) echocardiography criteria were used to assess LV and RV function and pressures. A right jugular central catheter was placed to assess central venous pressure (CVP) and ScVO2 in all patients. Right heart catheterization was not performed in this study. Systemic blood pressure was invasively monitored by an arterial line.

### 2.3. Management of ADHF

**Figure 1:**
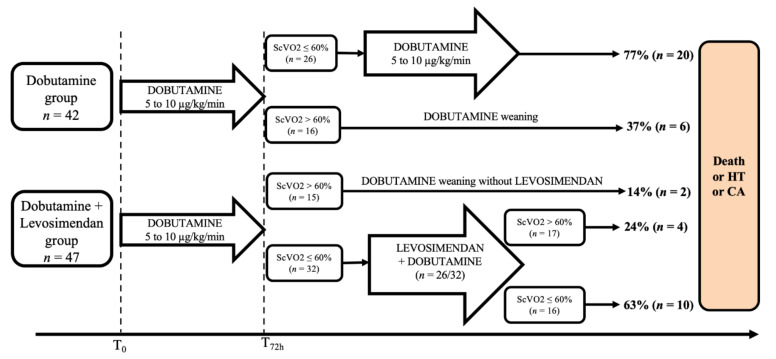
Flow chart of the population study. ScVO2: superior vena cava oxygen saturation, HT: heart transplantation; CA: cardiac assistance.

Patients admitted for ADHF with low cardiac output received a standard care according to our local protocol based on the current European Society of Cardiology guidelines for heart failure [8,9]. Intravenous loop diuretics (furosemide) were administered at a daily dose ranging from 500 to 1000 mg when CVP exceeded 10 mmHg. Furosemide dose was adjusted according to the urine output and the CVP. Diuretic dose was reduced by half if the daily urine output was > 4 L and was reduced to a maintenance dose if CVP was ≤10 mmHg, in order to achieve a neutral fluid balance. Dobutamine was initiated at a rate of 5 μg/kg/min and increased progressively (+ 2.5 μg/kg/min, maximum rate of 10 μg/kg/min) to a target ScVO2 value of >60%. If mean arterial blood pressure remained < 65 mmHg, norepinephrine was added at an initial rate of 0.5 mg/h. Dobutamine weaning was started after 72 h if the ScVO2 was > 60%. In the dobutamine group, dobutamine was administered for 48 to 72 additional hours if ScVO2 remained ≤ 60% and a slow weaning was attempted (0.1 μg/kg/min per hour). In the dobutamine-levosimendan group, levosimendan was added to dobutamine in patient’s refractory to dobutamine (ScVO2 ≤60% after 72 h of dobutamine). Levosimendan was administered without loading dose, at a rate of 0.2 μg/kg/min over 24 h (Figure 1). In case of low SBP (<90 mmHg), levosimendan infusion was started at a rate of 0.1 µg/kg/min during the first hour and increased to 0.2 µg/kg/min thereafter if well tolerated.

### 2.4. Outcome and Follow-Up

All patients aged <75 years were evaluated by the Heart Team for a potential need for mechanical circulatory support or heart transplantation in case of hemodynamic instability under inotropic support. All patients discharged alive from intensive care unit were referred to the heart failure team for treatment optimization and cardiac rehabilitation. The primary outcome of the study was defined as the difference in major cardiac events (MACE) at 6 months. MACE included all cause of death, emergency heart transplantation or need for mechanical circulatory support. Follow-up data were collected by patient or family contact or by reviewing patient’s electronic medical records.

### 2.5. Statistical Analysis

Nominal variables were expressed in percentage and were compared by the Chi-2 test or the Fisher exact test when necessary. Continuous variables with a normal distribution were expressed as mean ± standard deviation (SD) and were compared by a Student t-test or variance analysis. Paired analysis was used to assess changes in ScVO2 and an interaction term was computed to assess the impact of the therapeutic strategy. In patients treated with levosimendan, the final ScVO2 value used for outcome analysis was the one obtained 24 h after the end of levosimendan infusion. In the other patients, the final ScVO2 included in the analysis was the one assessed the day of dobutamine weaning. Non-normal distributed variables were expressed as median and inter-quartiles. Logistic regression and Cox model analysis were used to define variables associated with the outcome. Multivariate Cox analysis using stepwise regression was used to identify independent variables associated with the outcome. This is a pilot retrospective study with no previous data allowing calculating the number of subjects required. A *p*-value <0.05 was considered as statistically significant.

## 3. Results

Eighty-nine patients were included, 42 in the dobutamine-group and 47 in the dobutamine-levosimendan group. Baseline characteristics were similar between the two groups (Table 1).

**Figure 2:**
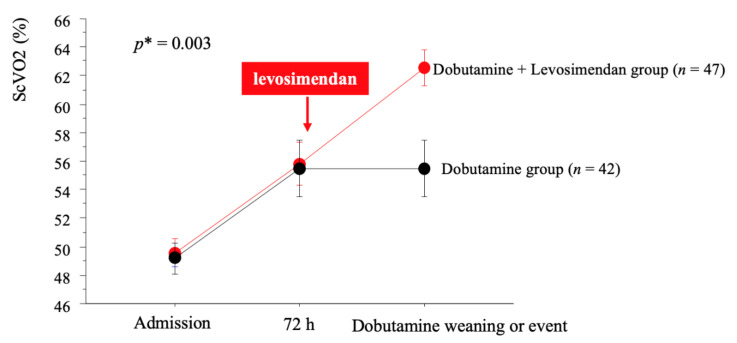
Changes in ScVO2 according to therapy strategy. *p* * indicates *p*-value for interaction term between changes in ScVO2 and therapy strategy. ScVO2: superior vena cava oxygen saturation.

Overall, left ventricle ejection fraction (LVEF) averaged 20 ± 6%, mean cardiac index and central venous pressure were 1.7 ± 0.6 L/min/m^2^ and 15 ± 6 mmHg, respectively. Prevalence of newly diagnosed LV dysfunction (*n* = 14) was similar between the dobutamine-levosimendan and the dobutamine group (17% vs. 14%, *p* = 0.72). In 44 patients (49%), the Heart Team stated that cardiac assistance or transplantation were not reasonable treatment options, mostly due to age, comorbidities or low expected treatment adherence. Implantable cardiac defibrillator was present in 37 patients (40% (*n* = 19/47) in the dobutamine-levosimendan group and 43% (*n* = 18/42) in the dobutamine group). Lactate level and ScVO2 at admission averaged 3.1 ± 2.6 mM/L and 50 ± 9%, respectively. Despite similar baseline characteristics, the dobutamine-group received higher doses of dobutamine at admission. Despite these differences, changes in lactate and ScVO2 during the first 72 h were similar between the two groups (Figure 2). Finally, the ScVO2 target (>60%) was reached in 34% of the total cohort, 38% (*n* = 16/42) in the dobutamine group and 32% (*n* = 15/47) in the dobutamine-levosimendan group (*p* = 0.55). The mean duration under dobutamine support was similar between both groups (6.8 ± 4 days vs. 6.6 ± 5 days *p* = 0.86).

### 3.1. Effect of Levosimendan

In the dobutamine-levosimendan group, 90% (*n* = 29/32) of patients with ScVO2 ≤60% under dobutamine were treated with levosimendan. Treatment was well tolerated in all but two patients; one patient presented hypotension and one patient presented acute pulmonary edema following levosimendan administration. ScVO2 increased from 50 ± 5% to 61 ± 7% (*p* < 0.01, Figure 2) the day after the end of the levosimendan infusion and CVP decreased from 10 ± 6 mmHg to 3 ± 3 mmHg (*p* = 0.005). Systolic blood pressure did not significantly change (101 ± 12 mmHg to 105 ± 12 mmHg), while diastolic blood pressure decreased (62 ± 9 mmHg vs. 56 ± 7 mmHg, *p* = 0.008). ScVO2 after levosimendan was >60% in 65% (*n* = 17/26) of patients and dobutamine dose was decreased from 5.0 ± 1.6 to 3.7 ± 2.7 μg/kg/min (*p* = 0.01) 24 h after the end of levosimendan infusion.

### 3.2. Outcome

**Figure 3:**
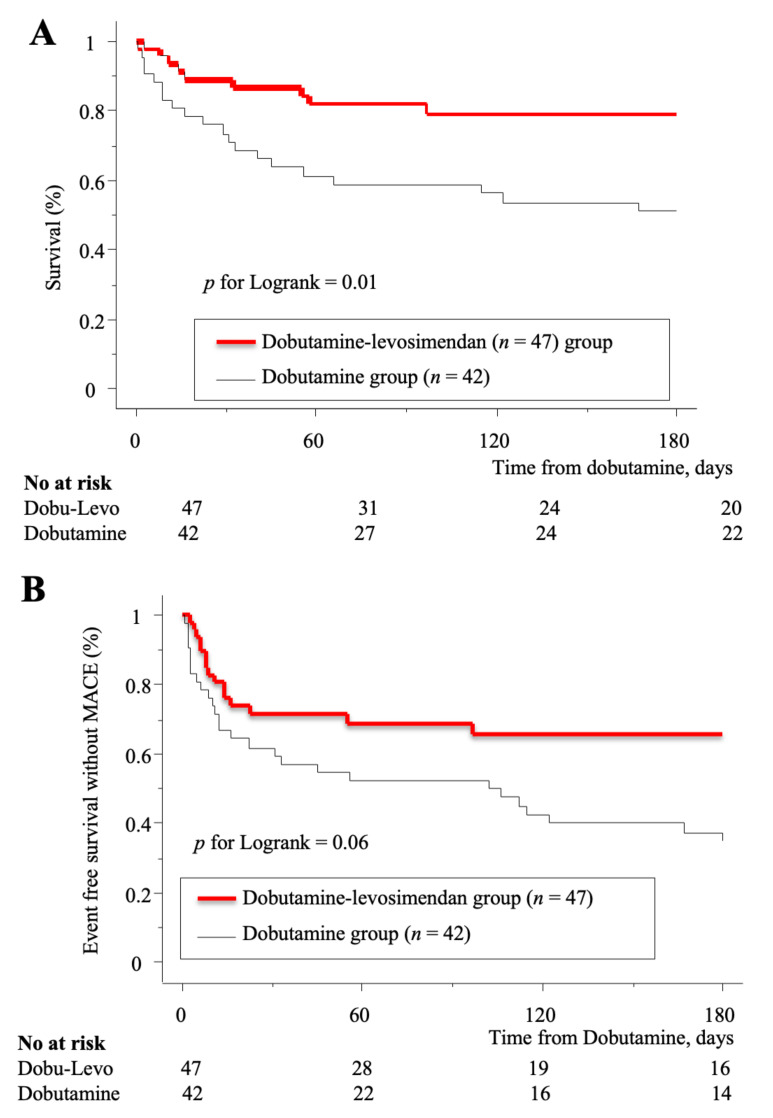
Kaplan-Meier analysis of event free survival without major cardiovascular events (MACE) (**A**) and death (**B**) according to the therapy strategy. ScVO2: superior vena cava oxygen saturation; MACE: major adverse cardiovascular events.

During the hospitalization period (26 ± 25 days), 18 died and 15 were referred for heart transplantation or mechanical circulatory support. There was a statistically non-significant trend towards a lower percentage of all causes death in the dobutamine-levosimendan group compared to the dobutamine group (12% vs. 29%, *p* = 0.06), while no difference was observed for heart assist devices and transplantation. Duration of intensive care unit (ICU) stay was similar between groups. Within the six-month period following dobutamine treatment, the cumulative risk of MACE (38% vs. 64%, *p* = 0.06) was lower in the dobutamine-levosimendan than dobutamine group (Figure 3).

**Figure 4:**
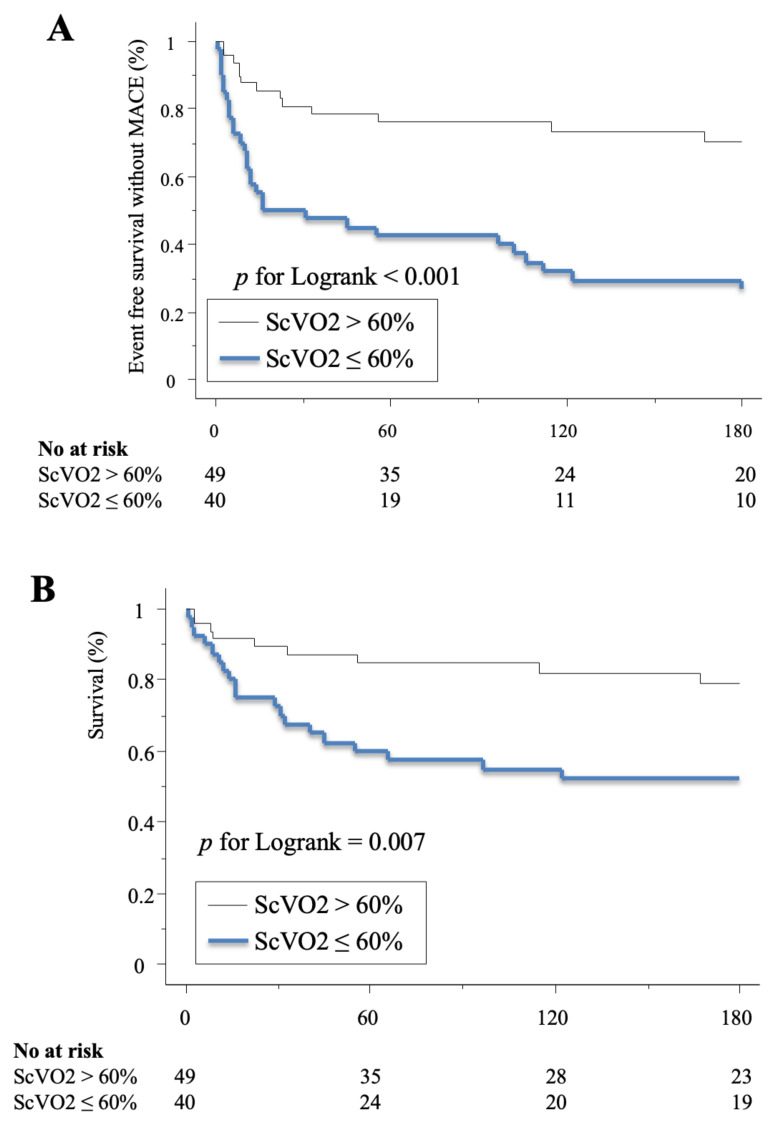
Kaplan-Meier analysis of event free survival without (**A**) and death (**B**) according to ScVO2 before event or dobutamine weaning. ScVO2: superior vena cava oxygen saturation; MACE: major adverse cardiovascular events.

Otherwise, the cumulative risk of death (21% vs. 49%, *p* = 0.007) and MACE (26% vs. 72%, *p* < 0.001) at 6 months were significatively lower for patients reaching a ScVO2 value of 60% at dobutamine weaning compared to those who do not (Figure 4).

Recurrent heart failure hospitalization (*n* = 11) did not differ between the two groups. Clinical variables associated with MACE from univariate analysis (cardiac index, blood pressures, furosemide dose, norepinephrine support and ScVO2 (Table 2)) were included in the multivariate analysis. In the model not including ScVO2, independent variables associated with MACE were admission SBP and dobutamine-levosimendan strategy (OR = 0.44 (0.23–0.84), *p* = 0.01, Table 3), whereas in the model including ScVO2, independent variables associated with MACE were the SBP and ScVO2 at the day of dobutamine weaning (OR = 4.3 (2.2–8.5), *p* < 0.001 Table 3).

## 4. Discussion

Management of patients with ADHF and low cardiac output remains a challenge, with only a few therapeutic options available. This study presents two important findings. Firstly, event-free survival following an episode of ADHF with low cardiac output is better when the target ScVO2 (>60%) is achieved. Secondly, treatment escalation by levosimendan infusion in dobutamine refractory patients, may improve clinical outcome.

ScVO2 as a tool to monitor critically ill patients has been well studied in septic shock, with one study showing a decrease in mortality with ScVO2 guided treatment [10], although subsequent studies failed to reproduce these results [11]. ScVO2 reflects the balance between oxygen consumption and supply. The prognostic value of ScVO2 has been addressed in different hemodynamic situations [12,13]. In CS and ADHF, the decrease in ScVO2 is not only caused by low cardiac output but also by increased oxygen consumption provoked by organ congestion. Gallet et al. [14] demonstrated that improving ScVO2 >60% in ADHF was associated with a reduction of in-hospital MACE. The present study demonstrates that this prognostic value extends up to a 6-month period.

ScVO2 can be increased by combining catecholamine treatment and aggressive diuretic strategy to decrease organ congestion [15,16,17]. Dobutamine is used as the first line inotrope support as recommended by the European Society of Cardiology (ESC) guidelines [8]. Dobutamine increases the myocardial contractility through adrenergic beta-receptor stimulation and may increase cardiovascular mortality by increasing oxygen consumption and myocardial apoptosis [1,2,3]. As an alternative, levosimendan is a calcium sensitizer which improves myocardial contractility without increasing oxygen consumption [4,18,19,20]. Levosimendan seems to improve hemodynamic performance and heart failure outcome in low-cardiac output patients [5]. However, the randomized Survive [6] study that compared levosimendan to dobutamine in ADHF did not show a difference in outcome. In a recent meta-analysis [21] including CS and ADHF related to a myocardial infarction, levosimendan was associated with an improvement in cardiac output and ScVO2 but mortality was unchanged compared to standard therapies or placebo. These results agree with recent randomized clinical trials [22,23,24] that failed to demonstrate a benefit in perioperative low-cardiac output syndrome. These data suggest that levosimendan and dobutamine have close inotropic efficiency and/or the pharmacological advantages of levosimendan cannot be translated into clinical benefit in moderately sized studies or in the perioperative setting. In our study, levosimendan was added to dobutamine to obtain a synergic effect in patient’s refractory to dobutamine. The staged increase in ScVO2 first after dobutamine and then after levosimendan infusion, illustrates the presence of two levels of contractile reserve, the first related to the beta-receptor stimulation and the second to the calcium sensitivity. Nanas et al. were the first to propose the combination of levosimendan to dobutamine [7]. Levosimendan plus dobutamine has shown to be superior to dobutamine alone for increasing cardiac output [25]. Our study is the first to be conducted in ADHF patients, demonstrating that this association is safe and efficient in terms of clinical outcome. A randomized trial (Levoheartshock, NCT #04020263) granted by the French Ministry of Health program (PHRC) will be conducted next year to assess the benefit of levosimendan on top of dobutamine in cardiogenic shock.

The main limitation of the study is the retrospective monocentric design that may bias the interpretation of the results. Indeed, dobutamine was more aggressively used in the dobutamine-group despite no difference in patient’s characteristics when compared to the levosimendan-dobutamine-group, which may induce a historical bias. This difference is related to an historical belief that aggressive inotropic support may be more efficient to restore hemodynamic stability. Nevertheless, these differences had been considered in the multivariate analysis. Of note, three patients in the levosimendan-dobutamine group did not receive levosimendan because of care limitation and family and physician decision.

## 5. Conclusions

In ADHF with low cardiac output requiring catecholamine support, levosimendan added to dobutamine seems to improve outcome of patients refractory to dobutamine.

## Figures and Tables

**Table 1:** Characteristics of patients treated with dobutamine or dobutamine plus levosimendan for cardiogenic shock.

Item	All (*n* = 89)	Dobutamine Alone (*n* = 42)	Dobutamine-Levosimendan (*n* = 47)	*p*
Age, years	61 ± 15	60 ± 16	62 ± 15	0.43
Sex, male, *n* (%)	64 (72)	29 (69)	35 (74)	0.64
LV dysfunction etiology				
CAD, *n* (%)	29 (33)	14 (33)	15 (32)	0.89
Dilated CM, *n* (%)	58 (65)	28 (67)	32 (64)	0.54
Others, *n* (%)	2 (2)	0 (0)	2 (4)	0.18
Diabetes, *n* (%)	24 (27)	14 (33)	10 (21)	0.24
Atrial fibrillation, *n* (%)	27 (30)	9 (21)	18 (38)	0.11
Newly diagnosed HF, *n* (%)	14 (16)	6 (14)	8 (17)	0,72
Care limitation, *n* (%)	39 (44)	17 (40)	22 (47)	0.67
Betablocker, *n* (%)	53 (60)	28 (66)	25 (53)	0.28
ACE inhibitor, *n* (%)	50 (56)	28 (60)	22 (53)	0.09
Aldosterone inhibitor, *n* (%)	46 (52)	23 (55)	23 (49)	0.67
CRT, *n* (%)	20 (22)	10 (24)	10 (21)	0.80
LVEF, %	20 ± 6	20 ± 6	19 ± 6	0.72
Cardiac Index, L/min/m^2^	1.7 ± 0.6	1.7 ± 0.5	1.8 ± 0.6	0.33
TAPSE, mm	12 ± 4	12 ± 4	12 ± 3	0.50
Admission				
SBP, mmHg	104 ± 20	103 ± 20	105 ± 19	0.71
DBP, mmHg	63 ± 14	61 ± 13	66 ± 13	0.11
HR, bpm	91 ± 20	90 ± 20	93 ± 20	0.50
Lactate, mM/L	3.1 ± 2.6	3.3 ± 3.0	3.0 ± 2.1	0.66
Creatinine, μM/L	154 ± 84	165 ± 84	143 ± 83	0.22
eGFR, mL/min	53 ± 28	48 ± 28	58 ± 29	0.11
Nt-proBNP, pg/mL	10,651 (5746–19,757)	12,930 (5641–26,691)	10,291 (5798–15,847)	0.22
ScVO2, %	50 ± 9	49 ± 7	50 ± 9	0.79
Dobutamine, γ/kg/min	6.8 ± 3.5	7.8 ± 4.3	5.8 ± 2.0	0.005
Furosemide, mg/h	30 ± 15	27 ± 15	32 ± 15	0.15
Mean norepinephrine, mg/h	0.9 ± 0.5	0.9 ± 0.5	0.9 ± 0.6	0.87
At 72 h				
SBP, mmHg	105 ± 16	104 ± 16	104 ± 16	0.80
DBP, mmHg	62 ± 12	61 ± 12	63 ± 11	0.42
HR, bpm	93 ± 21	92 ± 15	93 ± 24	0.88
Diuresis, L per day	3.9 ± 1.9	3.7 ± 2.0	4.0 ± 1.9	0.46
Dobutamine, γ/kg/min	6.6 ± 3.4	8.4 ± 3.9	5.0 ± 1.6	<0.001
Furosemide, mg/h	26 ± 16	21 ± 15	30 ± 16	0.007
Mean norepinephrine, mg/h	1.1 ± 0.7	1.2 ± 0.7	0.8 ± 0.5	0.34
Lactate, mmol/L	1.9 ± 0.6	2.0 ± 0.8	1.8 ± 0.5	0.13
ScVO2, %	56 ± 12	55 ± 13	56 ± 10	0.88
ScVO2 > 60%, *n* (%)	34 (36)	15 (36)	15 (32)	0.82
At dobutamine weaning or at MACE				
ScVO2, %	59 ± 11	54 ± 13	63 ± 9	0.003
ScVO2 > 60%, *n* (%)	49 (55)	15 (36)	34 (72)	0.03

Abbreviations: LV: left ventricular; CAD: coronary artery disease; dilated CM: dilated cardiomyopathy (idiopathic, toxic or post-myocarditis); Others: former valvular cardiopathy with functional prosthetic valve but persistent LV dysfunction; HF: heart failure; Care limitation indicates patients with a decision not to perform cardiac assistance or transplantation; ACE: Angiotensin Converting Enzyme; CRT: cardiac resynchronization therapy; LVEF: left ventricular ejection fraction; TAPSE: tricuspid annulus plane systolic excursion; SBP and DBP: systolic and diastolic blood pressure, respectively; HR: heart rate; eGFR: estimated glomerular filtration rate; NT-pro-BNP: N-Terminal pro Brain Natriuretic Peptid; H: hour; ScVO2: superior vena cava oxygen saturation; MACE: major adverse cardiovascular events.

**Table 2:** Univariate analysis of variables associated with MACE.

Item	MACE (*n* = 42)	Event Free (*n* = 47)	*p*
Age, years	61 ± 16	61 ± 15	0.96
Sex, male, *n* (%)	31 (74)	33 (70)	0.70
CAD, *n* (%)	14 (33)	15 (32)	0.89
Diabetes, *n* (%)	11 (26)	13 (28)	0.87
Atrial fibrillation, *n* (%)	11 (26)	16 (34)	0.42
Care limitation, *n* (%)	18 (43)	21 (45)	0.86
Beta-blocker, *n* (%)	27 (64)	26 (55)	0.74
ACE inhibitor, *n* (%)	24 (57)	26 (55)	0.86
Aldosterone inhibitor, *n* (%)	23 (55)	23 (49)	0.58
CRT, *n* (%)	13 (31)	7 (15)	0.08
LVEF, %	19 ± 6	20 ± 20	0.34
Cardiac Index, L/min/m^2^	1.6 ± 0.5	1.8 ± 0.6	0.06
TAPSE, mm	12 ± 4	12 ± 4	0.94
Admission			
SBP, mmHg	99 ± 14	109 ± 22	0.01
DBP, mmHg	60 ± 13	66 ± 13	0.04
HR, bpm	89 ± 18	93 ± 20	0.36
Lactate, mM/L	3.0 ± 2.6	3.2 ± 2.6	0.71
Creatinine, μM/L	169 ± 100	137 ± 62	0.10
Nt-proBNP, pg/mL	10,651 (5852–27,156)	10,651 (5534–19,394)	0.33
ScVO2, %	50 ± 7	49 ± 6	0.28
Dobutamine, γ/kg/min	6.7 ± 3.9	6.7 ± 3.9	0.98
Furosemide, mg/h	615 ± 387	800 ± 325	0.01
At 72 h			
SBP, mmHg	101 ± 10	108 ± 19	0.04
DBP, mmHg	60 ± 10	63 ± 13	0.23
HR, bpm	93 ± 15	92 ± 25	0.89
Diuresis, L per day	3.4 ± 1.8	4.4 ± 1.9	0.02
Dobutamine, γ/kg/min	7.1 ± 3.9	6.2 ± 2.8	0.21
Furosemide, mg/h	582 ± 373	652 ± 382	0.38
Norepinephrine, n (%)	10 (24)	3 (6)	0.03
Lactate, mmol/L	2.0 ± 0.6	1.8 ± 0.6	0.38
ScVO2, %	50 ± 11	60 ± 10	<0.001
ScVO2 > 60%	8 (19)	22 (47)	0.006
At dobutamine weaning or at MACE			
ScVO2, %	53 ± 12	65 ± 6	<0.001
ScVO2 >60%	13 (31)	36 (77)	<0.001

Abbreviations: LV: left ventricular; CAD: coronary artery disease; dilated CM: dilated cardiomyopathy (idiopathic, toxic or post-myocarditis); Others: former valvular cardiopathy with functional prosthetic valve but persistent LV dysfunction; HF: heart failure; Care limitation indicates patients with a decision not to perform cardiac assistance or transplantation; ACE: Angiotensin Converting Enzyme; CRT: cardiac resynchronization therapy; LVEF: left ventricular ejection fraction; TAPSE: tricuspid annulus plane systolic excursion; SBP and DBP: systolic and diastolic blood pressure, respectively; HR: heart rate; eGFR: estimated glomerular filtration rate; NT-pro-BNP: N-Terminal pro Brain Natriuretic Peptid; H: hour; ScVO2: superior vena cava oxygen saturation; MACE: major adverse cardiovascular events.

**Table 3:** Multivariate analysis of variables associated with MACE.

ScVO2 not Included in the Model	OR	*p*
Dobutamine-Levosimendan	0.44 (0.23–0.84)	0.01
Admission SBP	0.98 (0.96–0.99)	0.02
**ScVO2 included in the model**	**OR**	***p***
ScVO2 <60%	4.30 (2.20–8.50)	<0.0001
Admission SBP	0.98 (0.96–0.99)	0.01

Abbreviations: SBP: systolic blood pressure; ScVO2: superior vena cava oxygen saturation; MACE: major adverse cardiovascular events; OR: Odds Ratio.
